# The utility of transbronchial rebiopsy for peripheral pulmonary lesions in patients with advanced non-squamous non-small cell lung cancer

**DOI:** 10.1186/s12890-020-01277-6

**Published:** 2020-09-09

**Authors:** Akiko Tateishi, Yuji Matsumoto, Midori Tanaka, Toshiyuki Nakai, Shinji Sasada, Masahiro Aoshima, Takaaki Tsuchida

**Affiliations:** 1 Department of Endoscopy, Respiratory Endoscopy Division, National Cancer Center Hospital, 5-1-1 Tsukiji, Chuo-ku, Tokyo, Japan; 2 Department of Pulmonology, Kameda Medical Center, Kamogawa, Japan; 3 Cancer Medicine, Cooperative Graduate School, The Jikei University Graduate School of Medicine, Tokyo, Japan; 4 Department of Thoracic Oncology, National Cancer Center Hospital, Tokyo, Japan; 5 Department of Respiratory Medicine, Tokyo Saiseikai Central Hospital, Tokyo, Japan

**Keywords:** Diagnostic yield, Molecular profile, Rebiopsy, Transbronchial biopsy, Radial endobronchial ultrasound

## Abstract

**Background:**

Patients treated for non-squamous (non-Sq) non-small cell lung cancer (NSCLC) often require repeat biopsies to determine the optimal subsequent treatment. However, the differences between rebiopsy and initial biopsy in terms of their diagnostic yields and their ability to test the molecular profiles using bronchoscopy with radial endobronchial ultrasound guidance in patients with advanced NSCLC remain unclear. Hence, we aimed to compare the diagnostic yields and ability for molecular analyses of rebiopsies with those of initial biopsies.

**Methods:**

We investigated 301 patients with advanced non-Sq NSCLC who underwent radial endobronchial ultrasound-guided transbronchial biopsy (TBB) for peripheral pulmonary lesions (PPLs) between August 2014 and July 2017. Patients were divided into the rebiopsy and initial biopsy groups: the latter referred to the biopsy that determined the definitive diagnosis. The diagnostic yields and ability for molecular analyses were compared between the two groups, and the factors affecting the TBB diagnostic yield were identified using univariate and multivariate analyses.

**Results:**

The diagnostic yields of the rebiopsy and initial biopsy groups were comparable (86.8 and 90.8%, respectively; *p* = 0.287). Furthermore, 93.0 and 94.0% of the patients in the rebiopsy and initial biopsy groups, respectively, had adequate specimens for gene profiling and mutational analysis (*p* = 0.765). The factors that increased the diagnostic yield were a positive bronchus sign (*p* < 0.001) and tumour location within the internal two-thirds of the lungs (*p* = 0.026).

**Conclusions:**

The PPL diagnostic yield of the rebiopsy group was as high as that of the initial biopsy group. Hence, TBB for PPLs is feasible for patients requiring rebiopsy as well as for those with initial diagnoses. Adequate, high-quality biopsy specimens can be obtained by transbronchial rebiopsy for molecular testing.

## Background

Lung cancer is the leading cause of mortality worldwide [1, 2]. Cytotoxic chemotherapies such as platinum-based regimens have been the primary therapeutic option for advanced non-small cell lung cancer (NSCLC) [3]. However, recently developed molecular targeted therapies and immune checkpoint inhibitors have improved the survival of patients with corresponding gene mutations or a sufficiently high tumour proportion score (TPS) for programmed cell death ligand 1 (PD-L1) [4, 5]. Targeted therapies directed at tumour cells harbouring epidermal growth factor receptor *(EGFR*) gene mutations, anaplastic lymphoma kinase (*ALK*) gene rearrangements, ROS proto-oncogene 1 (*ROS1*) gene fusions, and B-Raf proto-oncogene (*BRAF*) gene mutations have produced impressive results [6–9]. One of the mechanisms underlying the resistance to *EGFR* tyrosine kinase inhibitors (EGFR-TKIs) is the threonine-to-methionine amino acid change at position 790 (i.e. T790M mutation) [10, 11]. The efficacy of osimertinib in T790M-positive patients with advanced NSCLC who experienced disease progression after receiving the first-line EGFR-TKI therapy was demonstrated in the AURA3 clinical trial [12]. However, a rebiopsy is required for detecting the T790M mutation in such patients [11, 13].

With the development of more advanced examination techniques, clinicians are employing novel diagnostic tests based on the individual’s genetics profile, other molecular mechanisms, and the TPS to establish a more personalised targeted therapy [14]. Next-generation sequencing (NGS) has been introduced to detect almost all the relevant gene mutations in patients with cancer in clinical practice [15, 16]; however, this procedure also requires obtaining adequate, high-quality biopsy specimens.

Rebiopsy in patients with non-squamous (non-Sq) NSCLC is important when selecting the subsequent lines of chemotherapeutic agents, EGFR-TKIs, or immune checkpoint inhibitors if their initial treatments are no longer effective. Previous studies have been performed to investigate rebiopsies for T790M analysis [17–20]; however, the differences between rebiopsy and initial biopsy in terms of diagnostic yields and their ability to test the molecular profiles using bronchoscopy with radial endobronchial ultrasound (R-EBUS) guidance have not yet been explored. Thus, this study aimed to compare the diagnostic yields obtained from rebiopsy to those obtained from initial biopsy (first diagnosis) and their ability to test the molecular profiles by means of bronchoscopy with R-EBUS guidance.

## Methods

### Patient selection

Data of consecutive patients who underwent R-EBUS-guided transbronchial biopsy (TBB) for peripheral pulmonary lesions (PPLs) at the National Cancer Center Hospital between August 2014 and July 2017 were reviewed. All patients with advanced non-Sq NSCLC were included in this study and were divided into a) the rebiopsy group (i.e. those who underwent repeat biopsies after receiving drug treatments) and b) the initial biopsy group (i.e. those who only had one biopsy for definitive diagnosis before receiving any treatments).

The clinical characteristics of the patients including age at the time of biopsy, sex, tumour size, location, and morphology (solid or part-solid), presence of bronchus sign, and visibility on chest radiography were recorded. The diagnostic yields and ability to test the molecular profiles were compared between the two groups; moreover, the factors affecting the diagnostic yield of TBB were identified using univariate and multivariate analyses. This study was approved by the National Cancer Center Institutional Review Board (no. 2018–090), and written informed consent was obtained from all patients.

### Bronchoscopy procedures

All bronchoscopy procedures were performed via the oral route under local anaesthesia. TBB procedures were carried out using any one of the following conventional flexible bronchoscopes (Olympus Ltd.): BF-1 T260, BF-260, BFP260, BF-F260, BF-1TQ290, BF-Q290, BF-P290, LF-TP, or BF-Y0053 [21]. A radial ultrasound probe (UM-S20-20R or UM-S20-17S; Olympus, Tokyo, Japan) was used in all cases; a guide-sheath (GS) kit (K-201 or K-203; Olympus Ltd., Tokyo, Japan) was used when warranted. A virtual bronchoscope was constructed using a workstation (Ziostation2; Ziosoft Ltd., Tokyo, Japan) to simulate the bronchial route that leads to the target PPLs [22]. A PPL was defined as an abnormal growth surrounded by the normal lung parenchyma that was not accessible with a conventional bronchoscope. Upon reaching the target bronchus, which leads to the PPL, the R-EBUS probe with or without a GS was inserted through the working channel of the bronchoscope and advanced towards the PPL under fluoroscopic guidance (VersiFlex VISTAVR; Hitachi Ltd., Tokyo, Japan). Ultrasonographic scanning was performed while manipulating the R-EBUS probe until the lesion was localised within the corresponding R-EBUS image. X-ray fluoroscopy (VersiFlex VISTA, Hitachi, Japan) was used in all cases to guide the insertion of the R-EBUS probe regardless of PPL visibility on chest radiography. The bronchus sign on computed tomography was defined as the presence of a bronchus leading directly to the PPL. Five or more specimens were collected from the patients as possible. The number of TBB specimens obtained from all cases was counted.

### Pathologic evaluation and mutational analysis

A positive diagnostic result obtained by TBB was defined as the identification of non-Sq NSCLC based on the histological and cytological features. The overall detection rate was determined on the basis of a positive diagnosis via histology and/or cytology.

*EGFR* mutation analyses of genomic DNA extracted from the tumour samples were performed using the Scorpion amplification-refractory mutation system. Additionally, the *ALK* rearrangements were analysed using immunohistochemistry (ALK iAEP® Nichirei Biosciences Inc., Tokyo, Japan) and fluorescence in situ hybridization (Vysis®, Abbott Laboratories, Abbott Park, IL, USA).

### Statistical analysis

The correlations between patient characteristics and biopsy success rate were analysed using the Fisher’s exact test for categorical variables. The comparison of R-EBUS detection rate between the two groups were analysed using a chi-squared test. A multivariate analysis using logistic regression was performed to determine the factors affecting the yield, although age and sex were excluded from the factors affecting the diagnostic yield. All tests were two sided, and a *p* value of < 0.05 was considered significant. All of the statistical analyses were conducted using the JMP 10 software (SAS Institute, Inc., Cary, NC, USA).

## Results

A total of 1763 patients underwent R-EBUS-guided bronchoscopy procedures for PPLs during the study period: among them, 301 were diagnosed with advanced non-Sq NSCLC. At least three specimens were taken from all patients. Five or more specimens were collected from 89.9% of the patients in the rebiopsy group and from 88.3% of those in the initial biopsy group.

**Fig. 1:**
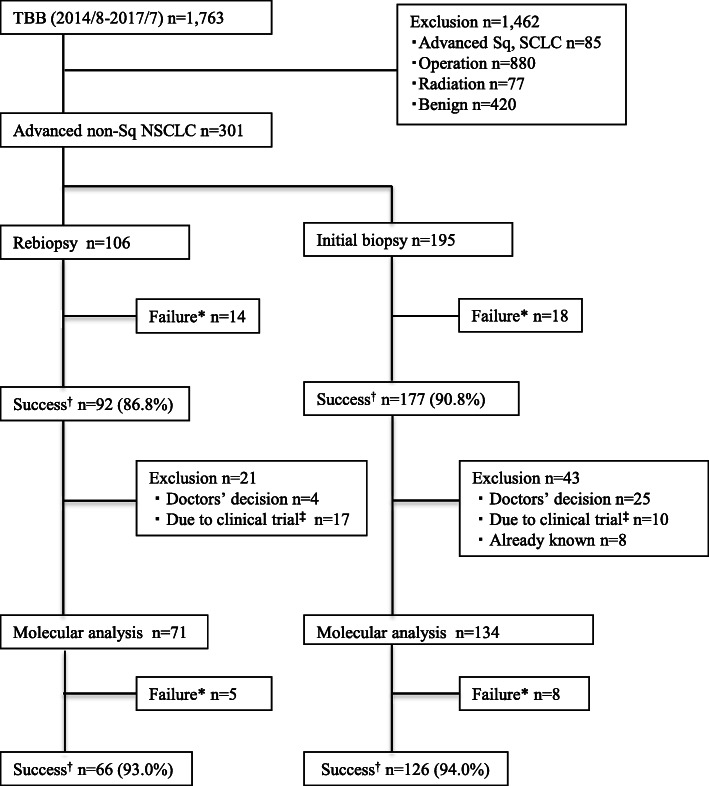
Flowchart of the study. The rebiopsy and initial biopsy groups were extracted and analysed according to the flowchart. *Failure, failed to pathological diagnosis; †Success, succeeded in pathological diagnosis; ‡Due to clinical trial, the purpose of biopsy was to collect specimens for the registration of clinical trials; NSCLC, non-small cell lung cancer; SCLC, small cell lung cancer; Sq, squamous; TBB, transbronchial biopsy

The rebiopsy and initial biopsy groups comprised 106 and 195 patients, respectively; their characteristics are listed in Table 1, and a flowchart of the study process is shown in Fig. 1. There were significant differences in the median ages of patients in the rebiopsy and initial biopsy groups (64 [range, 57–69] years vs. 68 [range, 59–75] years, *p* = 0.001) as well as sex (males, 38.7% vs. 53.8%, *p* = 0.012) and distance from the costal pleura (mm) (≥ 10.0, 44.3% vs. 31.8%, *p* = 0.031). No other significant differences were observed between the two groups, including tumour sizes, locations, and features, presence of bronchus sign, and visibility on chest radiography.

**Table 1:** Summary of patients’ clinical characteristics (*n* = 301).

Variables	Rebiopsy ***n*** = 106	Initial biopsy ***n*** = 195	***p***-value
Age, median (IQR)	64 (57–69)	68 (59–75)	0.001
Sex, no (%)			0.012
Male	41 (38.7)	105 (53.8)	
Female	65 (61.3)	90 (46.2)	
Lobe, no (%)			0.077
Upper	42 (39.6)	101 (51.8)	
Middle/Lingular	21 (19.8)	24 (12.3)	
Lower	43 (40.6)	70 (35.9)	
Location, no (%)			0.077
Internal 2/3	73 (68.9)	102 (52.3)	
External 1/3	33 (31.1)	93 (47.7)	
Distance from costal pleura (mm), no (%)			0.031
≥ 10.0	47 (44.3)	62 (31.8)	
< 10.0	39 (55.7)	133 (68.2)	
Size (mm), no (%)			0.845
≥ 30.0	74 (69.8)	134 (68.7)	
< 30.0	32 (30.2)	61 (31.3)	
Morphology, no (%)			0.053
Solid	79 (74.5)	124 (63.6)	
Part-solid	27 (25.5)	71 (36.4)	
Bronchus sign, no (%)			0.166
Positive	94 (88.7)	182 (93.3)	
Negative	12 (11.3)	13 (6.7)	
Visibility on chest radiography, no (%)			0.229
Visible	100 (95.2)	171 (91.4)	
Invisible	6 (4.8)	24 (8.6)	

IQR, interquartile range.

There was no significant difference between the rebiopsy and initial biopsy groups in terms of diagnostic yield (86.8% [92/106] vs. 90.8% [177/195]; *p* = 0.287). Moreover, no significant differences were found between the two groups in terms of lesion detection via R-EBUS: the rates of detection “within” the image for the rebiopsy and initial biopsy groups were 75.5% (80/106) vs. 73.9% (144/195), those of “adjacent to” were 22.6% (24/106) vs. 22.0% (43/195), and those of “invisible” were 1.9% (2/106) vs. 4.1% (8/195); *p* = 0.592 (Table 2). The factors affecting the diagnostic yield of TBB are shown in Table 3. In the multivariate analysis, the diagnostic yield was significantly higher for those with a positive bronchus sign (*p* < 0.001) and for those located in the internal two-thirds of the lungs (*p* = 0.026). By contrast, no significant difference was observed between the two groups depending on the purpose of rebiopsy (*p* = 0.400).

**Table 2:** Summary of diagnostic yield and R-EBUS detection (n = 301)

Variables	Rebiopsy n = 106	Initial biopsy n = 195	***p***-value
Diagnostic yield, no (%)	92 (86.8)	177 (90.8)	0.287
R-EBUS detection, no (%)			0.592
Within	80 (75.5)	144 (73.9)	
Adjacent to	24 (22.6)	43 (22.0)	
Invisible	2 (1.9)	8 (4.1)

**Table 3:** Univariate and Multivariate regression analyses of factors affecting diagnostic yield.

Variables	n (%) total 301	Univariate analysis	Multivariate analysis	
		*p*-value	*p*-value	Adjusted OR (95% CI)
Rebiopsy	106 (35.2)	0.287	0.400	–
Lobe		0.041	0.176	–
Upper	143 (47.5)			
Middle/lingular	90 (29.9)			
Lower	68 (22.6)			
Size ≥30.0 mm	208 (69.1)	0.016	0.311	–
Bronchus sign positive	276 (91.7)	< 0.001	< 0.001	132.6 (30.5–990.6)
Location in the internal 2/3 segments	175 (58.1)	0.015	0.026	4.6 (1.2–24.1)
Distance from costal pleura ≥10.0 mm	109 (36.2)	0.317	0.113	–
Visible on chest radiography	271 (90.0)	0.051	0.796	–

OR, odds ratio; CI, confidence interval.

Molecular profiles were evaluated in a proportion of cases (71 in the rebiopsy group and 134 in the initial biopsy group for *EGFR* mutation analysis, 8 in the rebiopsy group and 123 in the initial biopsy group for *ALK* rearrangements analysis). Adequate tumour samples were obtained from 93.0% (66/71) rebiopsy and 94.0% (126/134) initial biopsy patients for *EGFR* mutation analysis (*p* = 0.765), and from 100% (8/8) rebiopsy and 99.1% (122/123) initial biopsy patients for *ALK* rearrangements analysis (*p* = 1.000).

Although moderate bleeding (defined as 25–100 ml of blood) rates were relatively higher in the rebiopsy group than in the initial biopsy group (3.7% vs. 2.5%), none of the patients from both groups developed severe complications.

## Discussion

We conducted this study to compare the diagnostic yield of R-EBUS-guided TBB for PPLs between initial biopsy and rebiopsy after treatment. The diagnostic yield of R-EBUS-guided TBB in patients undergoing rebiopsy was as high as that in those undergoing initial biopsy (86.8% vs. 90.8%). In the multivariate analysis, the factors affecting the diagnostic yield of TBB were positive bronchus sign and tumour location within the internal two-thirds of the lungs. There was no significant difference between the two groups depending on the purpose of rebiopsy.

Previous investigations have found that the diagnostic yield of TBB using various modalities is approximately 70% [23–25]. Although no study has investigated the outcomes of patients with advanced non-Sq NSCLC, our diagnostic yield of 89.4% was considered sufficient. We believe that the high within-rate of R-EBUS detection contributed to our satisfactory diagnostic yield. The particularly large sizes of the tumours observed in advanced lung cancer, the high rate of positive bronchus sign (which are associated with tumour size), and the accurate guidance provided by some typess of navigation systems (achieved using a workstation), possibly contributed to the good results of R-EBUS detection [26].

Previous studies of patients undergoing rebiopsies produced TBB diagnostic yields of 73.2–80.7% [18, 20, 27]; however, these studies had a relatively small sample size and provided information only on patients undergoing rebiopsy. To our knowledge, no previous studies have compared the diagnostic data from rebiopsies and initial biopsies. In this study, we found that the diagnostic yield in the rebiopsy group was as high as that in the initial biopsy group with no significant difference (*p* = 0.287).

Previous studies revealed that tumour size, a positive bronchus sign, tumour location, and R-EBUS use are all factors that influenced the diagnostic yields [23, 24, 28]. Similar results were obtained in our study, with a positive bronchus sign and a tumour location in the internal two-thirds of the lungs producing higher diagnostic yields; moreover, a rebiopsy did not produce a significantly different diagnostic yield compared with an initial biopsy. Although rebiopsies are considered more difficult to perform than initial biopsies because of the increased tumour stiffness and haemorrhaging due to the administration of anticancer treatments, our results were not surprising because we used R-EBUS in all patients together with GS in those prone to bleeding. Wedging the GS in the target bronchus might help stop the bleeding after TBB [21]. Moreover, we used 30 mm as the cut-off tumour size as majority of the tumours were larger than the upper limit of T1 factor; the median length was 38 mm.

Two previous studies that investigated the feasibility of mutation analyses in rebiopsy specimens found that adequate tissue samples for mutational analyses were obtained from 74.4 and 89.7% of patients who underwent rebiopsies, respectively; the proportions of patients undergoing TBB for rebiopsy were 52 and 28%, respectively [29, 30]. In our study, 93.0% of successfully diagnosed patients in the rebiopsy group yielded adequate specimens for mutational analysis, while 94.0% in the initial biopsy group; the difference was not significant (*p* = 0.765). The high rate of successful mutational analyses in our study is likely related to the relatively large number of specimens obtained; at least five specimens were collected from 89.8% of the patients in the rebiopsy group and from 88.3% of those in the initial biopsy group.

Based on the results of the FLAURA trial, which demonstrated the efficacy of osimertinib as a first-line treatment for patients with *EGFR* mutation-positive NSCLC, this agent can be administered as a first-line therapy to eligible patients without requiring T790M mutation analysis beforehand [31]. However, many patients receiving first- and second-generation EGFR-TKIs will require rebiopsies when their cancers acquire resistance. Additionally, a rebiopsy is necessary not only for patients with *EGFR* mutation-positive NSCLC to detect T790M, but also for analysing *ALK* rearrangement, PD-L1 TPS, and aberrations in *BRAF* and *ROS1*, and to detect other genes including using NGS.

The collection of pathological specimens upon tumour progression following treatment with previous regimens can assist in understanding resistance mechanisms [32, 33]. Furthermore, new genomic mutations are continuously revealed along with the novel treatments that target them. Thus, rebiopsy still plays an important role in decision-making concerning subsequent-line treatments for patients with NSCLC. TBB is well-established as a minimally invasive method for lung biopsy, including rebiopsy.

This study has some limitations. First, the study was of a retrospective in nature. Second, the data were obtained from patients at a single institution that is specialises in cancer treatment. Third, the reasons for performing biopsies were multifactorial, in contrast to those in clinical trials where biopsies have a common purpose such as T790M detection or TPS evaluation. Hence, the assessments of mutations and of TPS were unsystematic. In addition, NGS had not been evaluated in all cases. However, selection bias was minimal, as this study reflects real-life clinical situations.

## Conclusions

The data from diagnostic bronchoscopic rebiopsies were comparable to those from initial biopsies. TBB for PPLs is a good an option for patients requiring rebiopsy as well as those requiring initial diagnostic biopsy. Adequate, high-quality biopsy specimens can be obtained by transbronchial rebiopsy for molecular testing.

## Data Availability

The datasets used during the current study are available from the corresponding author on reasonable request.

## Abbreviations

Non-Sq NSCLC: Non-squamous non-small cell lung cancer
TPS: Tumour proportion score
PD-L1: Programmed cell death ligand 1
EGFR: Epidermal growth factor receptor
ALK: Anaplastic lymphoma kinase
ROS1: ROS proto-oncogene 1
BRAF: B-Raf proto-oncogene
TKIs: Tyrosine kinase inhibitors
NGS: Next-generation sequencing
TBB: Transbronchial biopsy
R-EBUS: Radial endobronchial ultrasound
PPLs: Peripheral pulmonary lesions
GS: Guide-sheath
